# Biodistribution and scintigraphic evaluation of 99mTc-Mannan complex

**DOI:** 10.15190/d.2016.12

**Published:** 2016-10-01

**Authors:** Sweta Sanguri, Damodar Gupta, Thakuri Singh, Ajay K. Singh

**Affiliations:** Division of Metabolic Cell Signaling Research, Institute of Nuclear Medicine and Allied Sciences, Defence Research and Development Organization (DRDO), Brig SK Mazumdar Marg, Timarpur, Delhi 110054, India

**Keywords:** Mannan, Technetium-99m, scintigraphic imaging, non-invasive imaging, biodistribution studies, adjuvant

## Abstract

Technetium-99m (99mTc) is extensively used in nuclear medicine, mostly used to label radiopharmaceuticals and in radio diagnostics. In the present study, we directly radiolabeled mannan with 99mTc by using Tin(II) Chloride Dihydrate (SnCl2·2H2O) as a reducing agent. Mannan, a TLR agonist is a complex carbohydrate identified as a potential modulator of biological effects of ionizing radiation, both in vitro and in vivo, in our laboratory. Under in vivo conditions mannan modulates radiation response when administered through either oral or parenteral routes. The present study aims to understand the pharmacologic biodistribution of the 99mTc-mannan complex in mice (via oral, i.p. and i.v. routes) using non-invasive scintigraphic imaging and invasive radiometry. Qualitative and quantitative analysis of 99mTc-mannan complex was performed by ITLC-SG, ascending paper chromatography. Radio-complexation efficiency of >98% was consistently achieved with hydrolyzed reduced Tc-99m being 1-2%. We confirmed stability of complex in saline and serum up to 24 h at room temperature. Biodistribution studies were performed using the above radiocomplex in BALB/c mice and 99mTc-mannan complex was administered though oral, i.p. and i.v. routes. To our expectations, most of the radioactivity accumulated in stomach and small intestine in mice with oral administration, along with insignificant activity in the remaining studied organs. It suggests that 99mTc-mannan complex did not get absorbed from the gut and was removed as such in the fecal material. On the contrary, i.p. and i.v administration of mannan resulted in significant accumulation of the 99mTc-mannan complex in kidney, liver, intestine, lungs, spleen, bone marrow, blood and heart, at both 1 h and 4 h after i.v. administration. The remaining organs (stomach, testis and muscles) showed lower accumulation of the 99mTc-mannan complex. 99mTc-mannan complex was adminstered (i.v.) in New Zealand white rabbits and it was evident from the scintigraphic images that mannan cleared very rapidly from the administration site and reached into systemic circulation. No activity in the thyroid, salivary gland, or gastric mucosa suggests an insignificant amount of free pertechnetate in the 99mTc-complex preparation, further confirming the in vivo stability of the radiolabeled mannan complex. Significant amount of radioactivity in liver, intestine and kidneys suggests hepatobiliary as well as renal routes of clearance. The bio-availability of the complex varies with the route of administration. An entirely different biodistribution pattern exists when the same molecule is administered through oral or parenteral route. Our study is the first step towards a better understanding of the mechanisms involved in radiation modulation offered by mannan administration, in vivo.

## INTRODUCTION

^99m^Tc is an important short half-life radionuclide, first used for medical purposes in 1961^1^. ^99m^Tc has several features that make it safer than other available isotopes. Its short physical half-life (t_½ _6 h), biological half-life of 1 day (human activity and metabolism), low isotope cost, gamma energy of 140 KeV (same wavelength as emitted by conventional X-ray diagnostic equipment), easy availability in nuclear medicine laboratories, high sensitivity and quantitative measurements by medical equipment make it a potential candidate for imaging and functional studies *in vivo*^2,3^. ^99m^Tc can be incorporated into biomolecules directly or to the molecule that can be targeted against specific receptors or transporters *in vivo*. Various molecules, including cytokines, peptides, monoclonal and polyclonal immunoglobulins, and antibiotics, have been radio-labeled and are being used in non-invasive diagnosis or treatment of various ailments or diseases^4-7^.

Mannan, a TLR2 agonist has been explored for its radio-modifying efficacy at *in vitro* as well as *in vivo* level (oral and *i.p.* administration; unpublished data). The properties and pharmacological benefits of mannan are well characterized. Mannan has long been used as nutritional supplement in several living organisms that supports the gut microflora and has been shown to stimulate the immune system of the host^8-12^. It is known to increase microvilli surface area and goblet cell numbers in small intestine of mannan-supplemented animals^13,14^. It stimulates the immune system of the host and has adsorbent capacity against toxins and it is non-toxic when administered orally, even in large concentration^11,13,15-19^.

The purpose of this study was to investigate the bio-distribution pattern of mannan (from Saccharomyces cerevisiae) as it has been identified as a potential modulator of biological effects of ionizing radiation *via* various routes (*i.p., i.v. *and oral) of administration, *in vivo, *in our laboratory (unpublished data). Mannan is a complex carbohydrate and it is known to be resistant to digestion in animals, including human. Mannan exhibits radiation modulation efficacy, despite of the fact that it never reaches systemic circulation when taken orally. Moreover, similar efficacy has been sited with parenteral route, where mannan reaches systemic circulation directly. The results from the present study will help us to comprehend the mechanisms of radiation modulation offered by mannan administration, *in vivo*. ^99m^Tc-mannan complex used in this study was synthesized in laboratory. Mannan was labeled with Tc-99m by simple reduction method using stannous chloride dihydrate as a pertechnetate reductant. The labeling achieved was consistently more than 98%. The present study will provide us with a better understanding of the pharmacologic biodistribution of ^99m^Tc-mannan complex in various organs by non-invasive scintigraphic imaging, and invasive radiometry.

## MATERIALS AND METHODS

All the chemicals used in this study were of analytical grade unless otherwise specified. Human serum albumin, mannan (from Saccharomyces cerevisiae), stannous chloride, HPLC-grade solvents such as liquid ammonia, acetone, ethanol and all other reagents were procured from Sigma Chemicals Co, St Louis MO; USA. Tc-99m pertechnetate was procured from the regional center of BRIT, INMAS, Delhi. *In vivo* studies were carried out in male BALB/c mice and Male New Zealand rabbits. All animal experiments were conducted in accordance to institutional guidelines, and the Institutional Animal Ethical Committee.

### Radiolabeling of mannan and Quality control (ITLC-SG strips)

Mannan (2.5 mg) was dissolved in normal saline and mixed with 20 µg stannous chloride (SnCl_2_ 4 mg/ml in 0.01 N HCl solution). pH was adjusted to 7 with 0.01 N NaOH solution. ^99m^Tc (3 mCi) was added and mixture was incubated for 30 min. SnCl_2_ concentration and pH was optimized for maximum labeling efficiency (98.32 ± 0.9) and minimum colloids percent. All the labeling procedure was carried out in hot laboratory under lead shielding. Quantitative and qualitative assay of the ^99m^Tc-mannan complex was carried out by ITLC-SG (Agilent Technologies, CA, USA) ascending paper with two different mobile phases:100% acetone and ethanol : ammonia : water (1:2:5). Labeling efficiency of the complex was determined by separation of radioactivity into complexed (^99m^Tc-mannan complex), free (^99m^TcO_4_^-^), and reduced hydrolyzed technetium states (R/H ^99m^Tc).

### Stability of ^99m^TC-mannan radiocomplex

Stabilityof ^99m^Tc-mannan radiocomplex was assessed in saline and serum. 100 microliters of the radiolabeled complex were incubated in 1.9 ml of saline or human serum at 37°C. Small aliquots were withdrawn at different time intervals up to 24 h and radiolabeling efficiency was evaluated by ITLC-SG ascending paper with two different mobile phases:100% acetone and ethanol : ammonia : water (1:2:5).

### Biodistribution studies**

The biodistribution and excretory route of ^99m^Tc-mannan was studied in BALB/c mice (25–30 g; *n*=6). 100 μl of ^99m^Tc-mannan radiocomplex (1 mCi) was administered in mice by three different routes *viz.* intravenous, intraperitoneal and oral. Animals were sacrificed by cervical dislocation at 1h, 4 h and 24 h after ^99m^Tc-mannan administration. Organs of interest, namely, heart, liver, lungs, spleen, kidneys, stomach, intestine and muscle were removed, washed with saline and weighed, and respective radioactivity counts were determined with the help of a ϒ-counter (CAPRAC-R, Capintec, USA). Measured counts were adjusted to initial time with the respective decay of ^99m^Tc isotope. Data is expressed as percentage of administered radioactivity per gram of organ.

### Whole body scintigraphic studies

Gamma scintigraphy study was carried out using a dual head gamma camera system (Symbia T2, Siemens, Erlangen, Germany). Male New Zealand rabbits (n=3), weighing 2–3 kg were used in whole body scintigraphic studies. They were maintained on a normal diet. The animal was anesthetized by intramuscular administration of ketamine (15 mg/ kg). 2 mCi of ^99m^Tc-mannan complex was injected intravenously *via* ear vein. The anesthetized animal was placed in the supine position and scanned under the gamma camera. Anterior and posterior whole-body images were obtained after 10min, 2h, 4h and 24h.

### Statistical analysis

Data were expressed as mean ± SD of 6 mice per group. Results of bio-distribution experiments were statistically analyzed using Paired student’s t-test. Differences were considered statistically significant when *P *values were less than 0.05. All the data was analyzed using GraphPad PRISM, version 6.0 (GraphPad Software, San Diego, CA).

## RESULTS

### Radiochemical purity

Optimization of labeling efficiency of mannan was done by varying, conc. of stannous chloride and pH range. Radiochemical purity of ^99m^Tc-mannan was analyzed by ITLC-SG as stationary phase and acetone as mobile phase. In this system, ^99m^TcO_4_^-^ migrated with the solvent front of the mobile phase (Rf = 1.0) and the ^99m^Tc-mannan complex and colloids (R/H ^99m^Tc) were found at the origin of the strip (Rf = 0.3). Ethanol : ammonia : water (1:2:5) was used as mobile phase to segregate the radioactivity into complexed and colloidal states. In this system,^99m^Tc-mannan migrated with the solvent front of the mobile phase (Rf = 1.0) and the R/H ^99m^Tc was found at the origin of the strip (Rf = 0.2).

Radiochemical purity of ^99m^Tc-mannan, analyzed by ITLC-SG, was <98 ± 0.9%. Quality control tests using ITLC-SG revealed that ^99m^Tc-mannan contained less than 1% free ^99m^TcO_4_^-^ and 1–2% R/H ^99m^Tc (Table 1). The ^99m^Tc-mannan complex was stable at room temperature and there was no significant degradation of the complex up to 24 h.

**Table 1:** Radiochemical purity of of 99mTc–mannan complex Radiochemical purity of 99mTc–mannan complex was assessed by ITLC-SG with two different mobile phases: 100% acetone and Ethanol: ammonia: water (1:2:5). The results are expressed as percentage of mean ± SD of the respective components.

Radiochemical purity		
99mTc-mannan	99mTcO4-	R/H 99mTc
98 ± 0.9%	0.8 ± 0.42 %	1.2 ± 0.48 %

### In vitro stability of ^99m^Tc-mannan

**Figure 1:**
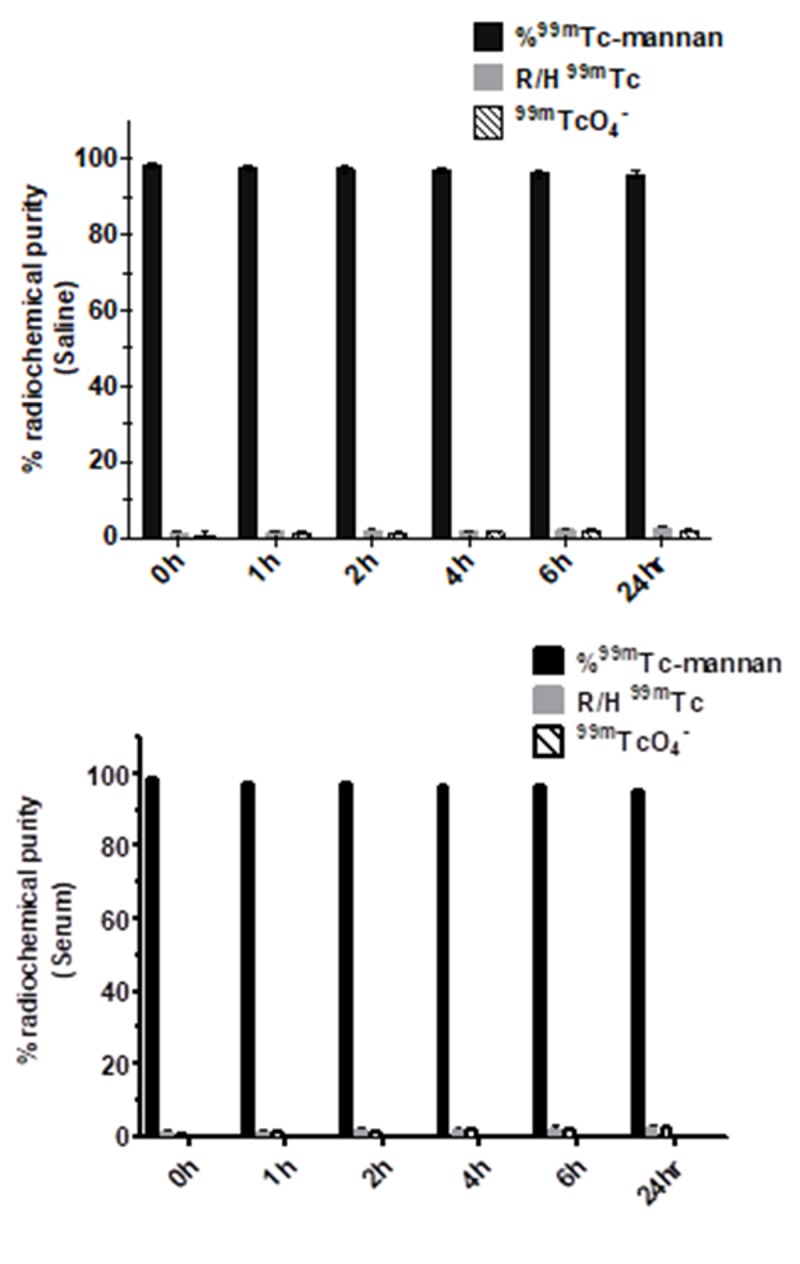
(A and B) In vitro stability of 99mTc–mannan in both normal saline and human serum: Stabilityof ^99m^Tc-mannan radiocomplex was assessed in saline and serum. The radiolabeled complex was incubated in saline or human serum at 37°C for different time intervals and small aliquots were withdrawn to measure radiolabeling efficiency using ITLC-SG with two different mobile phases:100% acetone and ethanol : ammonia : water (1:2:5). The results are expressed as percentage of radiochemical purity ± SD.

ITLC-SG analysis of the ^99m^Tc-mannan complex in saline (**Figure 1**[A]) and serum (**Figure 1** [B]) revealed that the ^99m^Tc-mannan complex remained sufficiently stable during incubation at 37°C with both saline and serum. A maximum of 4% of radioactivity degraded after 24 h of incubation advocating a high *in vitro* stability of almost 96% of the radiocomplex for up to 24 h.

### Biodistribution studies: i.v. and i.p. administration**

**Figure 2:**
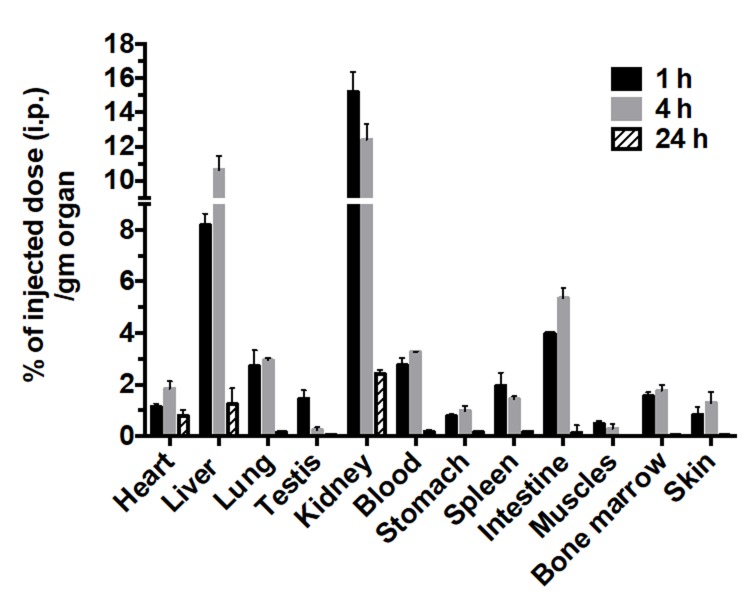
Biodistribution of 99mTc-mannan in mice following i.p. injection The biodistribution and excretory route of ^99m^Tc-mannan was studied in BALB/c mice. 100 μl of ^99m^Tc-mannan radiocomplex (1 mCi) was injected (*i.p*.). Animals were sacrificed at different time intervals after ^99m^Tc-mannan administration and respective radioactivity counts were determined in various organs as described in methodology section. Data is expressed as percentage of administered radioactivity per gram of organ ± SD.

**Figure 3:**
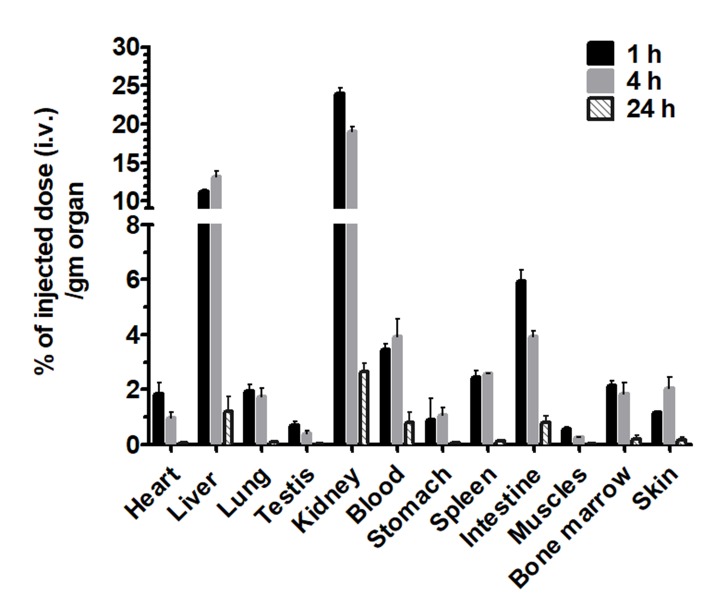
Biodistribution of 99mTc-mannan in mice following i.v. injection The biodistribution and excretory route of ^99m^Tc-mannan was studied in BALB/c mice. 100 μl of ^99m^Tc-mannan radiocomplex (1 mCi) was injected (*i.v*.). Animals were sacrificed at different time intervals after ^99m^Tc-mannan administration and respective radioactivity counts were determined in various organs as described in methodology section. Data is expressed as percentage of administered radioactivity per gram of organ ± SD.

Compartmental organ distribution of ^99m^Tc- mannan between 1 h to 24 h in healthy mice was studied (**Figure 2**and **Figure 3**). Prominent accumulation of the radiocomplex was observed in kidney (15.22±1.13, 12.40±0.91 and 2.41±0.15) and liver (8.18±0.45, 10.62±0.81 and 1.24±0.61) at 1 h, 4 h and 24 h respectively, followed by intestine, lungs, spleen, bone marrow, blood, heart and skin after *i.p.* administration of radiocomplex (**Figure 2**). Similar distribution pattern was observed in the organs after *i.v.* administration of radiocomplex with maximum accumulation in kidney (23.97±0.73, 18.98±0.61 and 2.65±0.31) and liver (11.26±0.25, 13.20±0.72 and 1.21±0.52) at 1 h, 4 h and 24 h respectively, followed by intestine, blood, spleen, skin, lungs, heart and bone (**Figure 3**). The study clearly indicates that the route of excretion of the radiocomplex is renal as well as hepatobiliary, since major accumulation was observed in kidneys and liver at all considered time intervals.

### Biodistribution studies:oral administration

**Figure 4:**
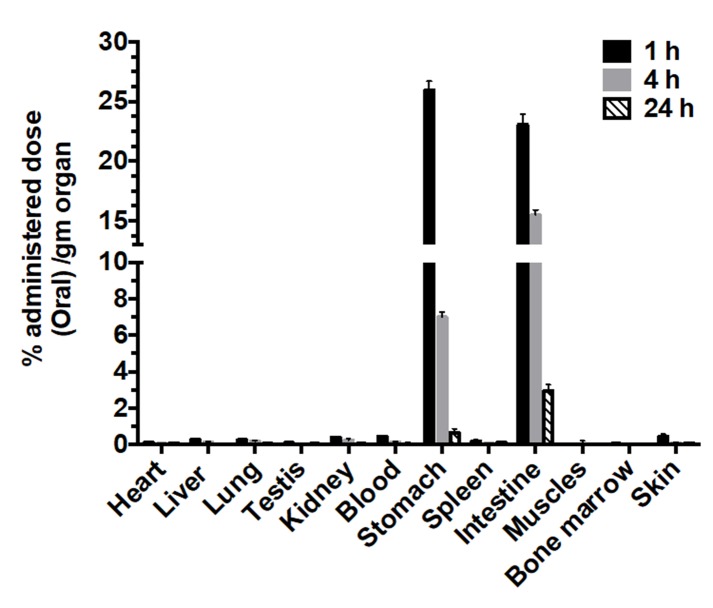
Biodistribution of 99mTc-mannan in mice following oral administration The biodistribution and excretory route of ^99m^Tc-mannan was studied in balb/c mice. 100 μl of ^99m^Tc-mannan radiocomplex (1 mCi) was administered (oral). Animals were sacrificed at different time intervals after ^99m^Tc-mannan administration and respective radioactivity counts were determined in various organs as described in methodology section. Data is expressed as percentage of administered radioactivity per gram of organ ± SD.

Biodistribution of ^99m^Tc- mannan administered orally in BALB/c mice is shown in **Figure 4**. Appreciable activity was noticed in the stomach and intestine at all the time intervals studied.

Moreover, insignificant radioactivity was observed in systemic circulation (kidney and bladder), and no significant radioactivity in remaining organs was a constant finding. Suggesting ^99m^Tc-mannan accumulated primarily in GI Tract and the major route of excretion of radiocomplex is intestinal.

### Scintigraphic images

**Figure 5:**
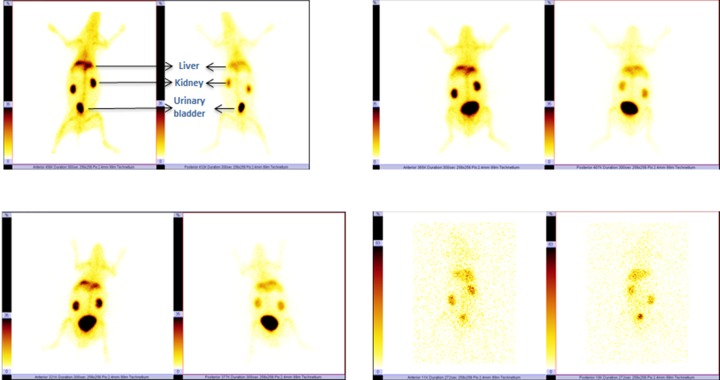
Whole body scintigraphic images of Mannan-99mTc complex Male New Zealand rabbits, weighing 2–3 kg were used in whole body scintigraphic studies. The animal was anesthetized by injecting ketamine (*i.m*.; 15 mg/ kg body weight). 2 mCi of ^99m^Tc-mannan complex was injected intravenously *via* ear vein. The anesthetized animal was placed in the supine position and scanned under the gamma camera. Anterior and posterior whole-body images were obtained after 10 min, 2 h and 4 h and subsequently at 24 h.

Localization of ^99m^Tc- mannan in normal healthy rabbits, as determined by gamma camera imaging, is shown in**Figure 5**. Renal and hepatic accumulation of radioactivity appeared soon after administration of radiocomplex (*i.v. *route). Significant urinary activity was also evident and kidneys were the main excretory organs. Significant renal cortex retention of radiocomplex was also a constant finding.

Bone or bone marrow activity was also evident. The biodistribution pattern seen on scintigraphic imaging with ^99m^Tc- mannan was similar to the data obtained by invasive radiometry after sacrificing the mice in different time intervals. All the injected animals survived the experiments and no clinical side-effects (*viz.* screaming, salivation, tremor, dyspnoe, diarrhoea, restlessness, or state of coma) were recorded in the animals. There was no sign of radioactivity in thyroid, salivary gland, or gastric mucosa at any time, indicating a high radioactive yield and stable *in vivo *labeling of mannan with ^99m^Tc.

## DISCUSSION

Mannan is a complex carbohydrate, included in diets in several living organisms including the farm animals, pigs, dogs, fishes, chicken, horses, cats, rabbits and birds due to its benefits for their health^11,13,15-17^. It is known to increase gut surface area and density *viz. *longer microvilli and shallower crypts, resulting in better digestion and absorption^13,14^. Increase in goblet cell numbers in small intestine of mannan-supplemented animals has also been reported^13^. Mannan has also been shown to posses’ antigenotoxic effect against aflatoxin B1, might be due to its adsorbent capacity^18^. Different studies have shown that salmonella binds to ma nnose *via* type-1-fimbriae (finger-like projections)^14^ which reduces the risk of pathogen colonization in the intestinal tract^20,21^. The protective activity of mannan has been demonstrated against the DNA damage induced by AFB1 in mouse hepatocyte. The potential preventive effect of the mannan against cancer development has also been shown^19^.

In the present study, we radiolabeled mannan**with ^99m^Tc to study its bio-distribution *in vivo*. Several important features, including short physical half-life (t_½_: 6h) and biological half-life (metabolism 24 h *in vivo*) of ^99m^Tc makes it one of the best radionuclides for imaging purposes. t_½ _of ^99m^Tc is long enough to complete the study and guarantees relatively low total radiation dose exposure to the experimental animals. Moreover, its gamma energy can be easily detected by a gamma camera, permitting the usage of moderately lesser quantities of radionuclide compared to other available isotopes^2,3^. In the present study, we radiolabeled mannan with ^99m^Tc and performed qualitative and quantitative analysis of labeled mannan by ITLC-SG, ascending paper chromatography. We confirmed the stability of complex in saline and serum up to 24 hrs at room temperature. Biodistribution studies were performed using the above radiocomplex in mice (BALB/c) after oral, *i.p.* or *i.v.* administration separately (**Figure 2**,**Figure 3**and **Figure 4**).

Mannan is a complex carbohydrate and it largely remains undigested in the gut. The human genome contains few of carbohydrate active enzymes that act on dietary carbohydrates. *Bacteroides spp* encodes a large number of carbohydrate active enzymes that can metabolize mannan. The genome of *Bacteroides thetaiotaomicron (Bt),* encodes 36 proteins possessing α-mannosidase or α-mannanase activity^22^. Depolymerization of complex, highly branched yeast mannan is an energy intensive process. Mannan is bulky in structure restricting the enzyme access, therefore its degradation requires a large number of enzymes^22^. The majority of dietary polysaccharides, including complex mannan is resistant to human digestion and simply remains undigested until it reaches colon. Conversely, an interesting biodistribution pattern endures when mannan is administered through parenteral route. *In vivo* accumulation of mannan depends on mannose receptor (MR) present in various organs and serum MBL (mannose binding lectin)^23^. MR is a carbohydrate-binding receptor expressed on kupffer cells (of liver) and macrophages (of spleen, alveolar and bone marrow)^24-27^. MBL is a multimeric pattern recognition protein of innate immunity, which is produced by liver in response to infection or specific foreign carbohydrate moieties, including terminal mannose^28,29^.

Among the various organs studied in animals with *i.p *and *i.v.* administration of radio-complex, significant accumulation of the radio-complex was found in kidney, liver, intestine, blood, lungs, spleen, bone marrow, heart and skin, at both 1 h and 4 h after administration (**Figure 2**and**Figure 3**). The radioactivity was significantly reduced at 24 h post administration, which could be due to elimination/ clearance of mannan from the body. Significant amount of radioactivity in the liver, intestine and kidneys suggest hepatobiliary as well as renal route of clearance for ^99m^Tc-mannan. Kupffer cells in liver express MR that has high affinity for mannan. Moreover, liver secretes MBL in blood serum in response to foreign carbohydrate, including mannan^23^. Serum MBL is highly specific for the carbohydrate against which it has been produced. The significant increase of radioactivity in blood might be because of presence of MBL in the serum, that specifically binds to ^99m^Tc- mannan. Affinity of serum MBL and ^99m^Tc- mannan complex might be one of the possible reasons of its renal route of clearance. The remaining organs (lungs, spleen and bone marrow) showed moderate accumulation of the ^99m^Tc- mannan complex, possibly because of the presence of alveolar, spleen and bone marrow macrophages respectively, that are known to expresses MR^23^. The radiometry data are similar to those obtained by scintigraphic imaging. High uptake of complex was observed in organs such as the liver, kidneys and urinary bladder. Hepatic accumulation of radioactivity appeared to increase with time, which probably reduced after 24 hours. It has earlier been shown that Candida mannan antigen gets cleared from the circulation mainly by the liver and spleen and to a lesser extent by renal filtration^30^. On the contrary, in the present study significant urinary activity was seen soon after administration of the radiocomplex and kidneys were the main excretory organs. Significant renal cortex retention of the radiocomplex was also a constant finding. Bone or bone marrow activity was also evident. There was no sign of radioactivity in the thyroid, salivary gland, or gastric mucosa at any time, indicating a high radioactive yield and *in vivo* stability of the ^99m^Tc- mannan complex. Quite the reverse, high uptake was observed is stomach and intestine in animals with oral administration of ^99m^Tc- mannan (**Figure 4**). The remaining organs including liver, kidney, heart, bone marrow and muscles exhibit insignificant uptake of the radiocomplex. It suggests that the complex did not get absorbed from the gut and egested in feces.

In summary, the present study demonstrates that mannan could be successfully labeled with ^99m^Tc with high radiolabeling efficiency and shows high and prolonged stability. Additionally, biodistribution and scintigraphic studies has been shown after ^99m^Tc- mannan complex administration by three different routes. Mannan has been identified as potential modulator of the biological effects of ionizing radiation, both *in vitro* and *in vivo,* in our laboratory (unpublished data). Under *in vivo* conditions, mannan modulates radiation response when administered through either oral or parenteral routes. Present study confirms that mannan is not absorbed from the gut and corroborates with available literature suggesting the lack of digestive enzymes to depolymerize mannan into subsequent monomeric residues. Mannan does not enter systemic circulation when administered orally, however an altogether different bio-distribution pattern exists for the same molecule when administered intraperitoneally. There might exist an entirely different mechanism of radiation modulation for both these routes of administration. The present study is the first step to better understand the mechanisms of radiation modulation offered by mannan administration, *in vivo*.

## Bullet Points

**◊ Oral administration of 99mTc- mannan in mice results in accumulation of radioactivity in stomach and intestine, confirming that mannan is not absorbed from the gut when administered orally and it is excreted by intestinal route**

**◊ i.v. and i.p. administrations of radiocomplex in mice shows compartmental distribution of the radiocomplex primarily in kidney, intestine and liver, exhibiting renal and hepatobiliary excretion**

**◊ The biodistribution pattern seen on scintigraphic imaging after intravenous administration of the radiocomplex in rabbit also shows renal and hepatic accumulation and bone marrow activity**
